# Molecular monitoring of *Plasmodium falciparum* resistance to artemisinin in Tanzania

**DOI:** 10.1186/1475-2875-5-126

**Published:** 2006-12-19

**Authors:** Kefas Mugittu, Blaise Genton, Hassan Mshinda, Hans Peter Beck

**Affiliations:** 1 Ifakara Health Research and Development Centre (IHRDC), P. O. Box 53, Ifakara, Tanzania; 2 Swiss Tropical Institute (STI), Socinstrasse 57, 4002 Basel, Switzerland

## Abstract

Artemisinin-based combination therapies (ACTs) are recommended for use against uncomplicated malaria in areas of multi-drug resistant malaria, such as sub-Saharan Africa. However, their long-term usefulness in these high transmission areas remains unclear. It has been suggested that documentation of the S769N *PfATPase6 *mutations may indicate an emergence of artemisinin resistance of *Plasmodium falciparum *in the field. The present study assessed *PfATPase6 *mutations (S769N and A623E) in 615 asymptomatic *P. falciparum *infections in Tanzania but no mutant genotype was detected. This observation suggests that resistance to artemisinin has not yet been selected in Tanzania, supporting the Ministry of Health's decision to adopt artemether+lumefantrine as first-line malaria treatment. The findings recommend further studies to assess *PfATPase6 *mutations in sentinel sites and verify their usefulness in monitoring emergency of ACT resistance.

## Background

Resistance to antimalarials is a major drawback in effective malaria control in sub-Saharan Africa. *Plasmodium falciparum *has developed high levels of resistance to the cheap and safe chloroquine (CQ) and to sulfadoxine-pyrimethamine (SP) [1,2]. Therefore, artemisinin-based combination therapy (ACT) is recommended for use in the whole of sub-Saharan Africa [3,4]. Fifteen out of 43 sub-Saharan African countries have already adopted artesunate + amodiaquine as first-line drug and the rest are at various stages of preparation for changes to the same regimen or to artemether + lumefantrine. Following increased SP resistance in Tanzania [5], the country has revised its malaria treatment policy to adopt artemether+lumefantrine. This change is supposed to be implemented over the whole country from December 2006. To date no relevant clinical resistance to artemisinins has been reported. However, the long-term usefulness of ACT in high transmission areas remains unclear [6]. Therefore, as ACT is becoming widely used in sub-Saharan Africa, the need for regular and comprehensive surveillance of resistance has been recognized. This should include *in vitro *and *in vivo *drug efficacy assays, pharmacokinetic analyses and the monitoring of molecular markers associated with resistance to the various components of ACTs.

Recently it was hypothesized, and later proved, that artemisinins interact [7] and selectively inhibit [8] PfATPase6, the only SERCA-type Ca^2+^-ATPase in the *P. falciparum *genome. A subsequent *in vitro *study in French Guyana showed that *P. falciparum *with elevated IC_50 _values for artemisinins shared specific point mutations at codon S769N of the *ATPase6 *locus. In addition, *ATPase6 *A623E and E431K mutations were also associated with reduced *P. falciparum *susceptibility to artemisinins [9]. This study suggested that documentation of these mutations may indicate emergence of *P. falciparum *artemisinin resistance in the field. Therefore, the aim of this study was to assess and determine the baseline prevalence of *ATPase6 *S769N and A623E mutations in Tanzania prior to adoption of ACT as first-line antimalarials drug.

## Methods

The study was conducted from July to November 2003 and 2004 in the Ipinda (south-west), Mlimba (south-east) and Mkuranga (east) health facilities in Tanzania. Malaria transmission in these rural areas is perennial with peaks between May and July. The study assessed asymptomatic infections (by *msp2 *amplification) in 1,205 randomly selected individuals (601 in 2003 and 604 in 2004) living in proximity to the health facilities. *P. falciparum *DNA from 615 *msp2 *positive samples was amplified by polymerase chain reaction (PCR) using *PfATPase6*-specific primary and nested primer pairs. The nested reaction spun a 798bp fragment of the *PfATPase6 *gene from which single nucleotide polymorphisms (SNPs) at positions 623 and 769 were detected by DNA microarray technique and a subset of PCR products were sequenced for quality control. This DNA microarray technique is designed to simultaneously detect the 32 reported antimalarial resistance conferring SNPs in *Pfdhfr*, *Pfdhps*, *Pfcrt Pfmdr1*, and *PfATPase6*. A detailed account of this technique has been prepared to be published separately (Crameri et al, submitted). In this article, only the recently described *PfATPase6 *SNPs, which are thought to decrease parasite susceptibility to artemisinin, are reported. The study was approved by the Tanzanian national and institutional review boards.

## Results

A total of 615 (51%) out of 1,205 individuals were *P. falciparum *positive by *msp2 *PCR amplification. 485 (75.6%) out of 615 had evaluable SNP data at positions 769 (n = 197) and 623 (n = 288) of the *PfATPase6 *gene. No *ATPase6 *S769N or A623E mutations were detected in any of the analyzed asymptomatic infection samples.

## Discussion

The study detected neither S769N nor A623E *PfATPase6 *mutations in any of the three sites including Mkuranga, a coastal region close to urban Dar es Salaam where a high rate of uncontrolled use of artemisinin monotherapy was reported [10]. The findings suggest that resistance to artemisinin has not been selected yet in Tanzania and are consistent with the high (~94%) artemether + lumefantrine parasitological cure rates recorded in a multi-country efficacy trial including Tanzania's coastal region [11]. These observations support the Ministry of Health decision to adopt artemether + lumefantrine as first line treatment for uncomplicated malaria. However, it is still unclear whether artemisinin-based combinations in high transmission areas of sub-Saharan Africa will retain their usefulness or robustness as did artesunate + mefloquine in low transmission areas of south-east Asia [6].

The increase in *P. falciparum multidrug resistance 1 *(*Pfmdr1*) copy number is believed to reduce parasite sensitivity to some quinolines antimalarials [12,13]. Therefore, *ATPase6 *genotype and *Pfmdr1 *copy number could make early warning signals for emergence of ACT resistance and enable investigators to timely advice the Ministries of Health on the most effective treatment strategies and appropriate policy-changing schedules. However, it should be noted that apart from one study [9], it has not been widely proven that SNPs in *PfATPase6 *confer resistance to artemisinins. Indeed one study [14] could not establish the function of *ATPase6 *in the artemisinin resistance of *Plasmodium chabaudi*. Thus, the role of *ATPase6 *in artemisinin resistance and its value in monitoring *P. falciparum *resistance to artemisinin remains to be confirmed.

## Conflicts of interests

The author(s) declare that they have no competing interests.

## Authors' contributions

K. Mugittu, B. Genton, H-P. Beck and H. Mshinda designed the study. K Mugittu performed molecular genotyping and data summarization. H. Mshinda supervised the field work in Tanzania. K. Mugittu wrote the article and all others contributed to it.

## Funding

This study was funded jointly by the European Union (Grant no. QLK2-CT-2002-01503, BBW 03.0001) and the Swiss National Foundation for Science (Grant no. 3100-067260). IHRDC receives core financial support from Swiss Agency for Development and Co-operation. Kefas Mugittu's PhD training programme is supported by Research Training Grants from the UNICEF/UNDP/World Bank/WHO Special Programme for Research and Training in Tropical Diseases (TDR).
